# A pectin-honey hydrogel prevents postoperative intraperitoneal adhesions in a rat model

**DOI:** 10.1186/s12917-017-0965-z

**Published:** 2017-02-17

**Authors:** Gessica Giusto, Cristina Vercelli, Selina Iussich, Andrea Audisio, Emanuela Morello, Rosangela Odore, Marco Gandini

**Affiliations:** Department of Veterinary Sciences, University of Turin, Largo Paolo Braccini, n. 2, Grugliasco, Turin, 10095 Italy

**Keywords:** Honey, Pectin, Hydrogel, Adhesion, Prevention, Rats

## Abstract

**Background:**

Adhesions are a common postoperative surgical complication. Liquid honey has been used intraperitoneally to reduce the incidence of these adhesions. However, solid barriers are considered more effective than liquids in decreasing postoperative intra-abdominal adhesion formation; therefore, a new pectin-honey hydrogel (PHH) was produced and its effectiveness was evaluated in a rat cecal abrasion model.

Standardized cecal/peritoneal abrasion was performed through laparotomy in 48 adult Sprague-Dawley rats to induce peritoneal adhesion formation. Rats were randomly assigned to a control (C) and treatment (T) group. In group T, PHHs were placed between the injured peritoneum and cecum. Animals were euthanized on day 15 after surgery. Adhesions were evaluated macroscopically and adhesion scores were recorded and compared between the two groups. Inflammation, fibrosis, and neovascularization were histologically graded and compared between the groups.

**Results:**

In group C, 17 of 24 (70.8%) animals developed adhesions between the cecum and peritoneum, while in group T only 5 of 24 (20.8%) did (*p* = 0.0012). In group C, one rat had an adhesion score of 3, sixteen had scores of 2, and seven rats had scores of 0. In group T, four rats had adhesion scores of 2, one rat had an adhesion score of 1 and nineteen have score 0 (*p* = 0.0003). Significantly lower grades of inflammation, fibrosis, and neovascularization were seen in group T (*p* = 0.006, *p* = 0.001, *p* = 0.002, respectively).

**Conclusion:**

PHH is a novel absorbable barrier that is effective in preventing intra-abdominal adhesions in a cecal abrasion model in rats.

**Electronic supplementary material:**

The online version of this article (doi:10.1186/s12917-017-0965-z) contains supplementary material, which is available to authorized users.

## Background

Postoperative intra-abdominal adhesion formation has long been considered an inevitable consequence of laparotomy, and the incidence in abdominal surgery is ranging between 67 and 93% [1–4]. Adhesion formation is a complex process involving cellular, biochemical, and immunological factors. Adhesions can develop when inflammatory responses perturb the equilibrium between fibrin formation and fibrinolysis [5]. The most common symptoms are chronic abdominal pain, small bowel obstruction, and secondary female infertility [3, 4, 6, 7]. These complications cause significant morbidity and pose a significant economic burden [6, 8].

Many potential preventive agents have been investigated, including intra-abdominal barriers, pharmacological agents, and synthetic devices [1, 4, 9]. At present, physical barriers are considered the most effective treatments for preventing postoperative intra-abdominal adhesion formation [10]. There are various types of commercially available anti-adhesion barriers such as hyaluronic acid/carboxymethyl cellulose membranes^1^ and hyaluronic acid/carboxymethyl cellulose gels^2^. These membranes work by preventing contact between damaged surfaces [3, 5, 10, 11].

In recent years, honey has been used for the prevention of postoperative peritoneal adhesions [5, 12, 13]. Honey is a heterogeneous substance that inhibits the growth of both gram-positive and gram-negative bacteria, has anti-inflammatory effects, and promotes healing processes following peritoneal damage [5, 12, 13, 14]. Because honey has proven effective in preventing intra-abdominal adhesions in liquid form, and since anti-adhesion barriers may be more effective than intraperitoneal solutions, we developed honey-based films that could be used as physical anti-adhesion barriers.

By combining some properties of honey such as hygroscopicity, viscosity, and healing effects with the properties of a physical barrier, we hypothesized that an effective means of preventing abdominal adhesions could be developed. Mixing honey with a gelling agent (pectin) resulted in the creation of pectin-honey hydrogels (PHHs) that can be easily applied between damaged organs in the peritoneum.

The aim of the present study was to evaluate the efficacy of PHHs for the prevention of intra-abdominal adhesions.

## Results

### Macroscopical examination

In 48 rats, no wound site infection or presence of intra-abdominal abscess was observed.

### Presence of adhesions

In group C, 17 of 24 (70.8%) animals developed adhesions between the cecum and the peritoneum, while in group T only 5 of 24 (20,8%) did (*p* = 0.0012) (Table 1).

**Table 1:** Presence of adhesions between cecum and peritoneum (*p* = 0,0012)

	Group C (*n* = 24)	Group T (*n* = 24)
Presence of adhesions	17	7
No presence of adhesions	5	19

In group C, 8 animals developed also adhesions between the omentum and linea alba, while in group T only 5 did. The difference in the two groups was not significant (*p* = 0.517).

In group C, 1 animal developed adhesion between the apex of cecum and the antimesenteric site of the distal jejunum, in group T 1 animal developed adhesion between the apex of the cecum and the side of a portion of mid-jejunum. In group T 1 animal developed adhesion between the apex of cecum and the median ligament of bladder (Table 2).

**Table 2:** Presence of other adhesions

	Group C (*n* = 24)	Group T (*n* = 24)
Omentum and linea alba	8	3
Cecum and small bowel	1	1
Cecum and bladder	0	1

### Adhesions scoring

In group C, one rat had an adhesion score of 3, sixteen had a score of 2, and seven had a score of 0. In group T, four rats had adhesion scores of 2, one rat had an adhesion score of 1 and nineteen have score 0 (*p* = 0,0003). Median adhesion score for group T was 2 (0,2), while for Group C was 0 (0,3). The distribution of scores in the two groups was significantly different (*p* = 0.0003) (Table 3).

**Table 3:** Median of the adhesion scoring

	Group C *n* =24	Group T *n* =24	*p* value
Adhesion scoring median (range)	0 (0,3)	2 (0,2)	0,0003

### Histopathological evaluation

The histopathological scores of the groups with respect to inflammation, fibrosis, and neovascularization are shown in Table 4. Significantly lower grades of inflammation, fibrosis, and neovascularization were seen in group T (*p* = 0.007, *p* = 0.001, *p* = 0.002, respectively).

**Table 4:** Median inflammation, fibrosis, and neovascularization scores

	Group C *n* =24	Group T *n* =24	*p* value
Inflammation score median (range)	1 (0,3)	0 (0,3)	0.006
Fibrosis score median (range)	1.5 (0,3)	0 (0,3)	0.001
Neovascularization score median (range)	3 (0,3)	0 (0,3)	0.002

Raw data are reported in an Additional file [see Additional file 1].

## Discussion

The PHHs developed in this study showed a local anti-adhesive effect that prevented intra-abdominal adherence in a cecal abrasion model in rats. In the control group, 17 of 24 rats developed adhesions between the cecum and peritoneum, while in treatment group only 5 of 24 rats did. The adhesion scores and the extents of adhesions were significant reduced after treatment with PHHs without complications related to tissue healing. Histopathological scores with respect to inflammation, fibrosis, and neovascularization were significant lower in the treatment group than those in the control group.

Many experimental models exist for studying peritoneal adhesions. The cecal abrasion model is a very effective way to create peritoneal adhesions because mechanical intestinal damage activates fibrinolysis and fibrinogenesis, leading to adhesion formation. The cecal abrasion model simulates the clinical situation of the viscera being manipulated to perform surgery. We modified this model by standardizing the number of passages with the gauze on the cecum and by assign animals to C or T group only after having performed the scraping. The first modification was made in order to standardize the damage inflicted to the cecal serosal. The second has been done in order to avoid a possible bias given by knowing in advance the group allocation of the specimen, thus influencing the surgeon toward performing less or more severe lesions.

Several researchers have used different scoring systems for grading adhesions [3, 12, 15, 16]. Many of these studies used Swolin’s scoring model, which macroscopically grades adhesions from 0 to 3 according to their severity. We chose to use this method because it is easy to apply and allowed us to express the results clearly [17].

In terms of methods of preventing adhesions, many studies have reported that pharmacological agents are not effective [10, 11]. Physical barriers are the most widely used method of adhesion prevention in humans, and have been shown to be effective in preventing intra-abdominal adhesions in animal models [10]. During recent years, many commercially available substances have been used to reduce the occurrence of postoperative adhesions. Carboxymethylcellulose bioresorbable membrane is one such material. Some studies have demonstrated that a carboxymethylcellulose-based membrane^1^ significantly reduces the incidence and extent of adhesions. However, the therapeutic effect of this membrane may be limited to the site of application, and the material seems difficult to handle in clinical practice [3, 5, 18].

In response to the need for effective solutions, honey has become increasingly popular and several studies have been published demonstrating its effectiveness in assisting the peritoneal healing process [5, 12]. However, studies regarding its effects on preventing peritoneal adhesions are still limited. Aysan and Emre used honey intraperitoneally to reduce postoperative adhesion formation, and reported that the treatment was effective and was associated with no toxic effects [5, 12].

In a preliminary unpublished in vivo test of the pectin-honey hydrogels, we treated three animals with PHH produced with 4 g of honey. All the animals died from ascites caused by the osmotic effect of the PHH. This fact was not reported by Aysan and Emre, and could have been caused by the presence of pectin in the films, the low content of water in the PHH, or the hygroscopicity of honey. In the second part of the study, we used 2 g of honey for PHH construction and none of the animals showed any side effects. This composition of PHH reduced inflammation and prevented adhesions formations without causing any side effects [19]. The results of this study demonstrated that PHH is effective in preventing the formation of postoperative adhesions. The material can be easily applied by surgeons and can be placed in deep anatomical sites. The PHHs are biocompatible and rapidly metabolized in contrast with the carboxymethylcellulose-polylactic acid membranes [18]. We hypothesize that the mechanical barrier combined with the absorption properties of honey may have played a key role in the inhibition of adhesion formation in the present study. More studies are needed on this topic, but the intraperitoneal use of PHHs does seem to significantly reduce the occurrence of postoperative adhesions at the site of application. PHH are not effective in preventing adhesion at distant sites from application, as demonstrated by the presence of adhesions between other organs other than cecum and peritoneum in treated animals. This is a limit of antiadhesion barriers, that extends to the product we tried in this study.

The present study was limited in one key way. We only used one model of adhesion formation, so we cannot be sure if the PHHs would behave the same if tested in a range of different models or even in genuine cases. Further, using dry gauzes to create the cecal abrasion, could be a confounding factor, because adhesions could be generated by small pieces of gauze lost during the procedures and acting as a foreign body on the surface of the intestine.

Another important limit is given by the use of a rat model. In fact, many agents that proved effective in rats couldn’t be demonstrated as effective in humans, and basing from this study we cannot predict PHH behaving differently.

## Conclusions

In conclusion, we observed that damaged cecal and abdominal wall surfaces treated with PHHs are significantly less likely to develop postoperative lesions. However, further investigations are needed in order to establish whether these results are transferable to other models and genuine cases, and to establish the true relevance of this treatment modality to the prevention of postoperative adhesions in the peritoneum.

## Methods

### Preparation of PHHs

We used a modified version of the preparation method described by Walker [19, 20]. Briefly, the PHHs were prepared from a starting solution (1:1 v/v) of liquid honey^3^ and sterile deionized water. Half this volume of pectin powder^4^ was then added gradually and with continuous stirring until the mixture was homogenized to obtain a final mixture of 1:1:1 v/v of honey, water and pectin. The resulting foam was spread into 2-mm films and hot-air-dried at 40 ± 0.5 °C for 6 h. We then cut the hard substance into 3 × 3-cm squares and further conditioned it in an air drier at 25 ± 1 °C for 5 d. The films were then collected and vacuum packed in polyethylene. All membranes were sterilized by gamma-irradiation at 25 kgray by Sterigenics International (Sterigenics International LLTC, Bologna, Italy) [19].

### Surgical procedures

All procedures were approved by the Bioethical Committee of the University of Turin and by the Italian Ministry of Health (protocol number 262/2015-PR on 20/04/2015).

A total of 48 adult male Sprague-Dawley rats weighting 225–250 g were purchased from Charles Rivers^5^ (Italy). All rats were housed in single cages for 7 d prior to the start of the experiment. The room temperature was set at 23 °C for the experimental period and the cages were cleaned daily. The rats were fed a commercial diet and water was provided *ad libitum*.

Anesthesia was induced by intramuscular administration of 5 mg/kg of xylazine^6^ and 50 mg/kg of tiletamine and zolazepam^7^. The rats we kept under anesthesia for 1 h, during which the abdomen was shaved and the skin was swabbed three times using an iodopovidone-chlorhexidine scrub.

A 4-cm midline incision was made in the abdominal wall. The cecum was exposed, and the apex was abraded with surgical gauze 100 times [21]. The left abdominal wall was abraded with a No. 21 scalpel blade 100 times. The cecum was returned to the abdominal cavity in its proper anatomic position. The scratching procedure was performed until hemorrhagic points were observed on the surface without perforation.

All surgeries were performed by the same operator (MG). Only after inducing injuries, the rats were randomly assigned to the treatment or control groups. In the treatment group, the PHHs were applied between the injured cecum and peritoneum. The midline incision was closed in two layers; the fascia was closed with 3-0-USP glycomer 631^8^, while the skin was closed with 3-0-USP nylon^8^. All surgical procedures took approximately 20 min. During the procedure and shortly thereafter, the rats were placed on a warm plate to avoid hypothermia, and 5-mL isotonic sodium chloride (0.9%) was administered subcutaneously. Animals were randomly divided into two groups of 24 animals each, using a free online calculator (www.random.org):

- Group C: negative control group. No treatment applied.
- Group T: group treated by applying the PHHs over the injury.

### Macroscopical examination

After 15 d, euthanasia was performed and the abdominal cavity was inspected through a U-shaped incision in the anterior abdominal wall. Signs of wound site infection or anastomotic abscesses were recorded.

### Presence of adhesions

Any macroscopic adhesion in the abdominal cavity, either involving the abrasion site or other organs were identified and recorded. Number of adhesions per group indicated as percentage were compared with a Fisher exact test.

### Adhesions scoring

Adhesions were scored according to the protocol described by Swolin [17] (Table 5). Scores were then compared between groups with a Mann-Whitney test.

**Table 5:** The adhesion grading by Swolin [17]

Grade	
0	No adhesions
1	Smooth adhesions that split either spontaneously or upon application of weak traction
2	Firm adhesions splitting when traction is applied
3	Dense adhesions requiring dissection with a blade

### Histopathological evaluation

The cecum and abdominal wall were harvested for histopathological examination and the tissue samples fixed in a 10% formaldehyde solution for histopathological examination. Samples were routinely processed by dehydration and paraffin embedding, and 5-μm cross-sections were produced. The samples were examined under a light microscope after hematoxylin-eosin staining and evaluated blindly by two expert pathologists to determine the presence of inflammation, fibroblastic activity, and neovascularization according to scores in accordance with Swolin [17] (Table 6).

**Table 6:** The presence of inflammation, fibroblastic activity, and neovascularization according to adherence scores by Swolin [17]

Grade	Inflammatory cell infiltrate	Fibrosis	Vascularization
0	Absent or normal in number	None	None
1	Slight increase	Slight	One to two vessels
2	Moderate infiltration	Moderate	Three to nine vessels
3	Dense	Dense	Ten or more vessels

### Statistical analysis

Sample size was calculated for the parameter “Presence of adhesions” hypothesizing for group 1 75% and for group 2 25% of animals with adhesions. Alpha level was set at 0.050 and Power at 89%. Further 4 animals were added to the result to account for possible deaths in the operative or postoperative period. Statistical analyses were performed with GraphPad Prism 6 software^9^. The normality of the data was evaluated using the Shapiro–Wilk normality test. Non parametric tests were used for not-normally distributed data. A *p* < value 0.05 was considered statistically significant.

## Additional file

Additional file 1: Raw data of adhesions necropsy and histological scoring. (PDF 99 kb)

## Abbreviation

PHH: Pectin-honey hydrogel

## Footnotes

1 Sanofi S.p.A, Milano, Italy

2 Hanmi Medicare, Songpa-gu, Seoul, South Korea

3 DermaSciences, Princeton, NY

4 ARDET s.r.l., Cuneo, Italy

5 Charles Rivers, Lecco, Italy

6 Bayer Animal Health, Milano, Italy

7 Virbac, Milano, Italy

8 Covidien, Segrate Milano, Italy

9 GraphPad Software Inc, La Jolla, USA
