# Supplementary material for: A pectin-honey hydrogel prevents postoperative intraperitoneal adhesions in a rat model
Source: BMC Vet Res. 2017 Feb 17;13:55. doi: 10.1186/s12917-017-0965-z (PMC5314697; doi:10.1186/s12917-017-0965-z)
Supplement: Additional file 1: — Raw data of adhesions necropsy and histological scoring. (PDF 99 kb) [file 12917_2017_965_MOESM1_ESM.pdf]

	Control	Treated
**Adhesion**	17	5
**No adhesion**	7	19

Adhesions scoring

Control	Treated
0	0
0	0
0	0
0	0
0	0
0	0
0	0
2	0
2	0
2	0
2	0
2	0
2	0
2	0
2	0
2	0
2	0
2	0
2	0
2	1
2	2
2	2
2	2
3	2

Extent adhesion (mm^2)	
Control	Treated
100	200
48	100
48	30
100	100
400	160
100	0
160	0
100	0
30	0
30	0
225	0
200	0
50	0
300	0
100	0
100	0
200	0
0	0
0	0
0	0
0	0
0	0
0	0
0	0

Grades of fibrosis

Control	Treated
0	0
0	0
0	0
0	0
0	0
0	0
1	0
1	0
1	0
1	0
1	0
1	0
2	0
2	0
2	0
2	0
2	0
2	0
2	1
3	1
3	1
3	3
3	3
3	3

Grades of inflammation

Control	Treated
0	0
0	0
0	0
0	0
0	0
0	0
0	0
0	0
0	0
0	0
1	0
1	0
1	0
1	0
1	0
1	0
1	0
1	0
2	0
2	0
2	0
2	3
3	3
3	3

Grades of Neovascularization

Control	Treated
0	0
0	0
0	0
0	0
0	0
0	0
0	0
0	0
2	0
2	0
2	0
3	0
3	0
3	0
3	0
3	0
3	0
3	0
3	0
3	2
3	3
3	3
3	3
3	3
